# Impact of SLCO1B3 767G>C (rs60140950) polymorphism on paclitaxel response in Iraqi women with breast cancer

**DOI:** 10.1016/j.jtumed.2026.08.002

**Published:** 2026-08-24

**Authors:** Balsam S.J. Al-Hussein, Atheer M.R. Al-Juhaishi, Hassan M.M. Abo Almaali, Ahmed A.J. Alaskari

**Affiliations:** a Department of Pharmacology and Toxicology, College of Pharmacy, University of Kerbala, Karbala, Iraq; b Department of Clinical Pharmacy, College of Pharmacy, University of Kerbala, Karbala, Iraq; c Department of Clinical Laboratory Sciences, College of Pharmacy, University of Kerbala, Karbala, Iraq; d Clinical Oncologist in Oncology Department, Imam Hassan Al-Mujtaba Hospital, Kerbala, Iraq

**Keywords:** SLCO1B3, تعدد الأشكال الجيني, الباكليتاكسيل, سرطان الثدي, Breast cancer, Genetic polymorphism, Paclitaxel, SLCO1B3

## Abstract

**Objective:**

The aims of this study were to identify genetic polymorphism in solute carrier organic anion transporter 1B3 (SLCO1B3 767G>C (rs60140950)), and to examine the impacts of this polymorphism on the efficacy and safety of paclitaxel in Iraqi women with breast cancer (BC).

**Methods:**

This cross-sectional observational study involved 150 women with BC who were administered paclitaxel. During the second week of paclitaxel therapy, these women were evaluated individually using a questionnaire to gather demographic information, such as their age and body mass index, as well as the likelihood and intensity of adverse effects of paclitaxel. Neutropenia levels and BC biomarkers were also evaluated.

**Result:**

The wild-type genotype of the SLCO1B3 767G>C genetic polymorphism was identified in approximately 76% of BC cases. The mutant type (CC) and heterozygous type (GC) were identified in approximately 10% and 14% of cases, respectively. Among Iraqi women with BC carrying GG, GC, and CC genotypes, no significant differences were found in BC markers (cancer antigen 15-3 (CA 15-3) and carcinoembryonic antigen (CEA)) or paclitaxel-related side effects (neutropenia, oral mucositis, and peripheral neuropathy) at *P* > 0.05.

**Conclusion:**

The distribution of the SLCO1B3 767G>C (rs60140950) genotype was high among Iraqi women with BC. No significant correlations were found between the SLCO1B3 767G>C genetic variant and changes in BC markers (CA 15-3 and CEA) or the incidence of early paclitaxel-related adverse effects.

## الملخص

**أهداف البحث:**

هدفت هذه الدراسة إلى تحديد تعدد الأشكال الجيني SLCO1B3 767G>C (rs60140950)، ودراسة تأثيره على الاستجابة للباكليتاكسيل وسلامته لدى النساء العراقيات المصابات بسرطان الثدي.

**طرق البحث:**

أُجريت دراسة رصدية مقطعية شملت 150 امرأة مصابة بسرطان الثدي تلقين العلاج بالباكليتاكسيل. وخلال الأسبوع الثاني من العلاج، جُمعت البيانات الديموغرافية، بما في ذلك العمر ومؤشر كتلة الجسم، إضافة إلى تقييم حدوث الآثار الجانبية المرتبطة بالباكليتاكسيل وشدتها. كما تم تقييم العدلات وقياس مؤشرات سرطان الثدي CA 15.3 وCEA. وحدد تعدد الأشكال الجيني SLCO1B3 767G>C باستخدام تفاعل البوليميراز المتسلسل الخاص بالأليل (AS-PCR).

**النتائج:**

أظهر التحليل الجيني أن النمط الجيني البري GG كان الأكثر شيوعاً، إذ وُجد لدى نحو 76% من المشاركات، في حين بلغت نسبة النمط غير المتماثل GC نحو 14%، والنمط المتماثل المتغير CC نحو 10%. ولم تُظهر النتائج فروقاً ذات دلالة إحصائية بين حاملات الأنماط الجينية GG وGC وCC فيما يتعلق بمؤشري سرطان الثدي CA 15.3 وCEA أو بالآثار الجانبية المرتبطة بالباكليتاكسيل، بما في ذلك نقص العدلات والتهاب الغشاء المخاطي للفم والاعتلال العصبي المحيطي (P > 0.05).

**الاستنتاج:**

أظهرت الدراسة وجود تعدد في الأنماط الجينية للمتغير SLCO1B3 767G>C (rs60140950) لدى النساء العراقيات المصابات بسرطان الثدي. ومع ذلك، لم تُظهر النتائج وجود ارتباط ذي دلالة إحصائية بين هذا المتغير الجيني والتغيرات في مؤشرات سرطان الثدي CA 15.3 وCEA أو حدوث الآثار الجانبية المبكرة المرتبطة بالعلاج بالباكليتاكسيل ضمن النتائج التي تم تقييمها في هذه الدراسة.

## Introduction

Breast cancer (BC) is the most prevalent cancer globally, accounting for one in four cases among women, and is the most commonly diagnosed disease in most nations.^1^ BC is an extremely lethal cancer that constitutes around 34.35% of all cancer cases among Iraqi women.^2^^,^^3^ BC is characterized as complex due to differences in genetic predisposition, environmental influences, aging, lifestyle choices, and dietary factors. In the past 10–15 years, treatment paradigms have progressed to address this variability, focusing on enhancing the therapeutic efficacy and minimizing the side effects of chemotherapy.^4^^,^^5^ BC biomarkers, such as cancer antigen 15-3 (CA 15-3) and carcinoembryonic antigen (CEA), are commonly utilized to assess prognosis and to evaluate the efficacy of chemotherapy in women with BC.^6^ Taxanes comprising paclitaxel and its semi-synthetic derivative docetaxel have become conventional therapies for multiple solid tumors, including breast, lung, ovarian, and prostate cancers. Their therapeutic impact is achieved by disrupting microtubular activity through binding a cytotoxic chemical to the β-subunits of tubulin. This interaction facilitates microtubule assembly and stabilizes microtubule dynamics, ultimately leading to mitotic arrest and cell death.^7^ Resistance has been noted in some cases.^8^ Several researchers have studied the reasons for this resistance and demonstrated the roles of many factors, such as the cytochrome P450 system inactivating paclitaxel,^9^ modification of cancer cell cycle regulation via the overproduction of microtubulin-related proteins,^10^ and expression of adenosine triphosphate-binding cassette transporters.^11^ Paclitaxel resistance remains a critical therapeutic problem because many women with BC are on paclitaxel therapy. Another problem with paclitaxel therapy is serious adverse effects, including neutropenia, peripheral neuropathy, alopecia, and oral mucositis.^12^ The severity of these side effects may be consistent with the decreased efficacy of paclitaxel. The causes of this phenomenon remain unclear and further studies are required to identify and overcome them.^13^ The solute carrier organic anion transporter family member 1B3 (SLCO1B3) gene encodes SLCO1B3, or organic anion-transporting polypeptide family member 1B3 (OATP1B3), a transmembrane transport protein within the OATP superfamily. These transporters enable the cellular uptake of a wide array of endogenous and exogenous organic anions. OATP1B3 is mostly localized on the basolateral (sinusoidal) membrane of hepatocytes within the liver.^14^ OATP1B3 mediates the uptake of many drugs by the liver, thereby influencing their pharmacokinetics, and its substrates include taxane, methotrexate, imatinib, statins, and other compounds.^15^ Functional studies have demonstrated that certain SLCO1B3 coding variants, including Gly256Ala, may modify the substrate affinity or transport efficiency, although the clinical impact appears to be limited.^16^ In vitro functional studies have demonstrated that SLCO1B3 767G>C rs60140950 variants can modify the abundance of transporters, substrate selectivity, and pH-modulated function, but with no significant effect on the transport capacity for atorvastatin.^17^ A study involving 190 American Caucasian and African-American cases showed that the pharmacokinetics of paclitaxel were not significantly associated with SLCO1B3 genetic variation in Caucasian cancer populations.^18^ The aims of the present study were to identify the genetic polymorphism in active transporter SLCO1B3 767G>C (rs60140950), and to examine the impacts of this polymorphism on the efficacy and safety of paclitaxel in Iraqi women with BC.

## Materials and Methods

### Patients

Iraqi women aged 50–78 years with BC were diagnosed in accordance with the National Comprehensive Cancer Network clinical practice guidelines.^19^ The women were randomly selected inpatients from the oncology department of Imam Al-Hussein Medical City following the acquisition of consent. Women were eligible for this trial if they received paclitaxel as monotherapy at a dosage of 80 mg/m^2^, provided via 1-h intravenous infusion weekly for a minimum of two cycles, without any concurrent illnesses. Women were excluded with cardiovascular diseases, diabetes mellitus, hepatic dysfunction, hyperthyroidism, and those on medications that influence the pharmacokinetic and pharmacodynamic properties of paclitaxel, including ketoconazole, erythromycin, rifampin, phenytoin, abaloparatide, abametapir, abatacept, and abciximab.

### Study design

This cross-sectional observational study was conducted from December 2024 to March 2025, and involved 150 women with BC who received paclitaxel, where the final sample size was determined using Fisher's formula.^20^ The original target population comprised approximately 242 women with BC who received paclitaxel, as identified by the oncology department at Imam Al-Hussein Medical City. The women were evaluated individually during the second week of paclitaxel medication by using a questionnaire to gather demographic information, including age and body mass index (BMI), as well as the likelihood and intensity of side effects due to paclitaxel. Neutropenia levels and BC biomarkers were also evaluated.

### Blood collection

Approximately 6 mL of blood was extracted from each woman and partitioned into two segments, where 2 mL was allocated to an ethylenediaminetetraacetic acid tube for absolute neutrophil count (ANC) and genetic analyses, and 4 mL was placed in a gel tube to obtain serum for evaluating BC markers.

### Measurement of BC markers

The serum levels of CA 15-3 and CEA were quantified using the chemiluminescent microparticle immunoassay technique^21^ with an ARCHITECT i1000SR immunoassay analyzer and CA 15-3 and CEA kits from Abbott, USA.

### White cell count

A differential complete blood count was performed to evaluate hematological parameters, i.e., ANC, which is a vital sign for assessing bone marrow function and chemotherapy-induced myelosuppression in women with BC who are undergoing paclitaxel treatment. The test was conducted using an automated Swelab Alfa Hematology Analyzer (Boule Diagnostics AB, Sweden).

### Assessment of severity of paclitaxel-induced neutropenia

The severity of paclitaxel-induced neutropenia was typically assessed using a scale based on the Common Toxicity Criteria established by the National Cancer Institute. This measure categorizes neutropenia into four categories depending on the ANC: score 1, ANC ≥1.5 to <2 × 10^9^/L; score 2, ANC ≥1.0 to <1.5 × 10^9^/L; score 3, ANC ≥0.5 to <1.0 × 10^9^/L; and score 4, ANC <0.5 × 10^9^/L.^22^

### Assessment of paclitaxel-induced oral mucositis

Paclitaxel-induced oral mucositis was evaluated using the World Health Organization (WHO) scale, which integrates clinical assessment with functional and symptom-based evaluations of oral mucositis. This scale defines oral mucositis as follows: score 0, no oral mucositis; score 1, erythema and soreness; score 2, ulcers, capable of consuming solids; score 3, ulcers, necessitating a liquid diet; and score 4: ulcers, alimentation not feasible.^23^

### Assessment of paclitaxel-induced peripheral neuropathy

Peripheral neuropathy generated by paclitaxel was assessed based on the WHO Common Toxicity Criteria for Peripheral Neuropathy, which employs a five-point scoring system: score 0, absence of neuropathy symptoms; score 1, paresthesia (tingling, tickling, or prickling feeling) and/or diminished tendon reflexes; score 2, severe paresthesia and/or mild weakness; score 3, intolerable paresthesia and/or significant motor loss; and score 4, paralysis.^24^

### Genotyping

Genomic DNA was isolated from whole blood samples utilizing an AddPrep™ Genomic DNA Extraction Kit (AddBio, Korea) in accordance with the manufacturer's guidelines. The allele-specific polymerase chain reaction (AS-PCR) method was utilized to identify SLCO1B3 767G>C (rs60140950) gene polymorphisms with a Mastercycler Gradient thermal cycler (Eppendorf, Germany). This method has been extensively employed for single-nucleotide polymorphism (SNP) genotyping due to its superior specificity and economic efficiency in differentiating alleles based on single-nucleotide variation.^25^ For each SNP, two allele-specific forward primers (one for each allele) and one common reverse primer were constructed according to Dr. Hassan Abo Almaali. The primer sequences for this gene were: forward (allele G) 5′-AAGGACTCTCGTTGGGTTGG-3′, forward (allele C) 5′-AAGGACTCTCGTTGGGTTGC-3′, and reverse 5′-GCAGAGAAGAAAGCTGACTCTA-3′, with a product size of 337 bp. Each PCR reaction was conducted in a total volume of 25 μL comprising 3 μL of DNA template, 2 μL of each forward and reverse primer, 5 μL of 5 × Master Mix (AddBio, Korea), and 13 μL of distilled water. The PCR parameters for rs60140950 consisted of initial denaturation at 95 °C for 5 min, followed by 35 cycles comprising 25 s at 95 °C, 25 s at 55 °C for annealing, and 1 min at 72 °C, and then a final extension at 72 °C for 5 min. After amplification, 5 μL of each PCR product was combined with 3 μL of loading dye and applied to a 1.5% agarose gel prepared with 1 × TBE buffer and 0.5 μg/mL ethidium bromide. Electrophoresis was conducted at 100 V for 35 min. DNA bands were observed under ultraviolet light with a transilluminator and recorded using a digital camera. Band sizes were determined by comparison with a 100–1000 bp DNA ladder. All outcomes were validated by two independent evaluators.

### Statistical analysis

The Statistical Package for the Social Sciences (SPSS 26) was employed for statistical analyses. The Shapiro–Wilk test was applied to assess the normality of data distributions. Numerical data were reported descriptively as the mean ± standard error of the mean for normally distributed data, and the median ± interquartile range for non-normally distributed data. Qualitative data were represented as numerical values and percentages. Normally distributed data, such as age and BMI, were compared by using one-way analysis of variance with the post hoc least significant test, whereas non-normally distributed data were analyzed using nonparametric tests, i.e., the Kruskal–Wallis test via legacy dialogs in SPSS. Non-numerical data, including paclitaxel-associated side effects, were compared utilizing the Chi-square test. The distribution of SLCO1B3 767G>C gene alleles was examined utilizing the Hardy–Weinberg equation, nonparametric testing, legacy dialogs, and the Chi-square test. *P* values below 0.05 were considered to indicate statistically significant differences.

## Results

### Demographic data for BC women

Analysis of demographic data for women with BC indicated a mean age of 52.02 ± 0.87 years and BMI of 29.36 ± 0.42 kg/m^2^, respectively.

### Serum levels of BC markers

The median serum concentrations of BC markers were 17.95 for CA 15-3 and 2.37 for CEA.

### Incidence and severity of paclitaxel-related adverse effects

Paclitaxel-induced neutropenia was detected in all women with BC, with variable levels of severity, and a score of 3 was predominant. Most women with BC who received paclitaxel experienced mucosal erythema, tenderness, and feeding difficulties, where scores of 1 and 2 were most prevalent among these patients. Paresthesia, weakness, and diminished tendon reflexes were identified as prevalent symptoms of peripheral neuropathy in women with BC after paclitaxel treatment, where scores of 1 and 2 were frequently diagnosed in these patients, as shown in Table 1.

**Table 1:** The incidence and severity of paclitaxel-related adverse effects.

Paclitaxel-related adverse effects		No (%)
Neutropenia	Score 1	25 (16.6%)
	Score 2	24 (16%)
	Score 3	82 (54.7%)
	Score 4	19 (12.7%)
Oral mucositis	Score 0	32 (21.4%)
	Score 1	50 (33.3%)
	Score 2	59 (39.3%)
	Score 3	9 (6%)
	Score 4	0 (0%)
Peripheral neuropath	Score 0	9 (6%)
	Score 1	70 (46.7%)
	Score 2	55 (36.7%)
	Score 3	13 (8.6%)
	Score 4	3 (2%)

Data expressed as No (%).

### Genotyping

#### Prevalence of SLCO1B3 767G>C (rs60140950) gene and allele distribution

**Figure 1:**
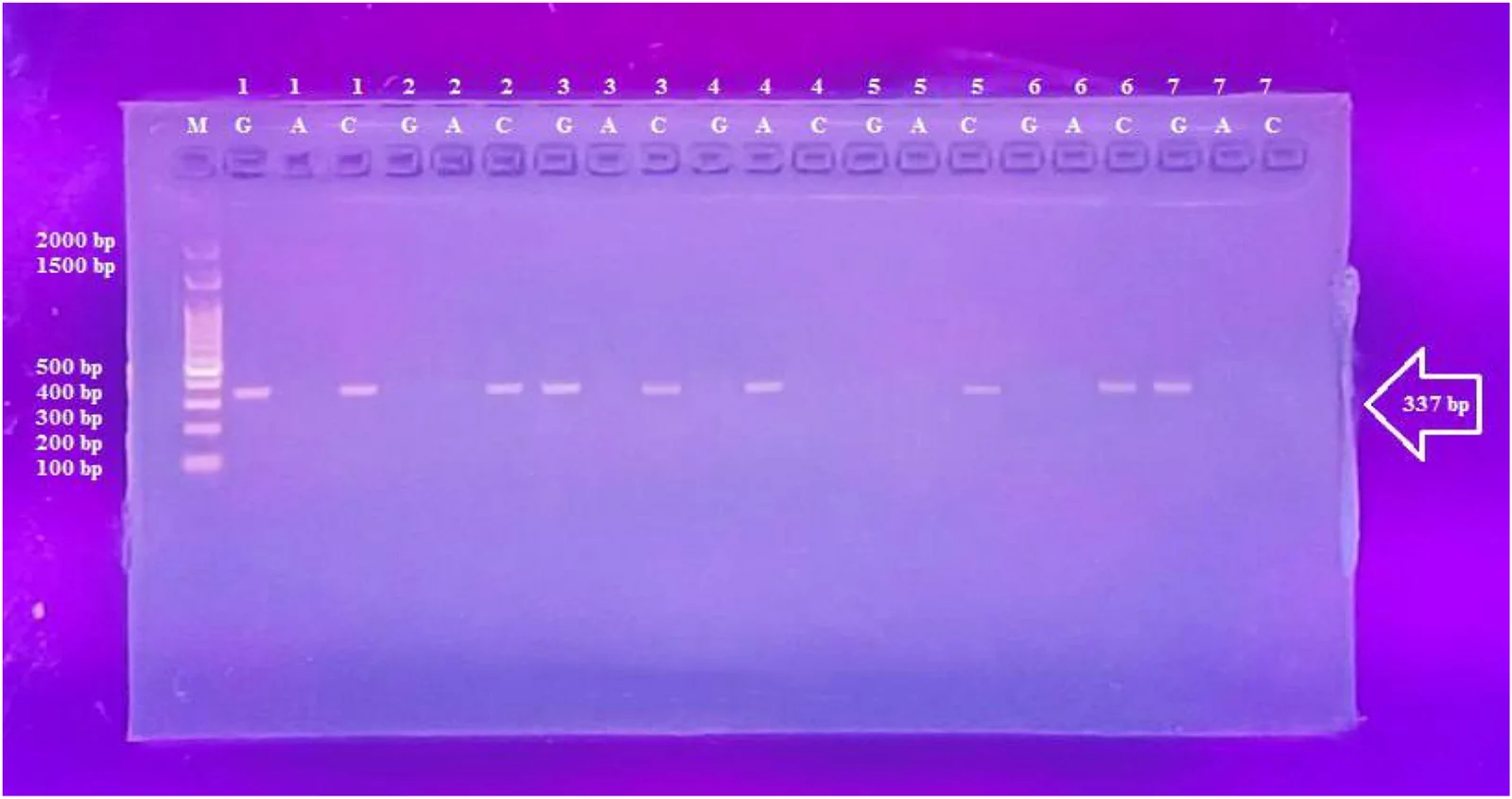
Genotyping of SLCO1B3 767G>C gene.

**Figure 2:**
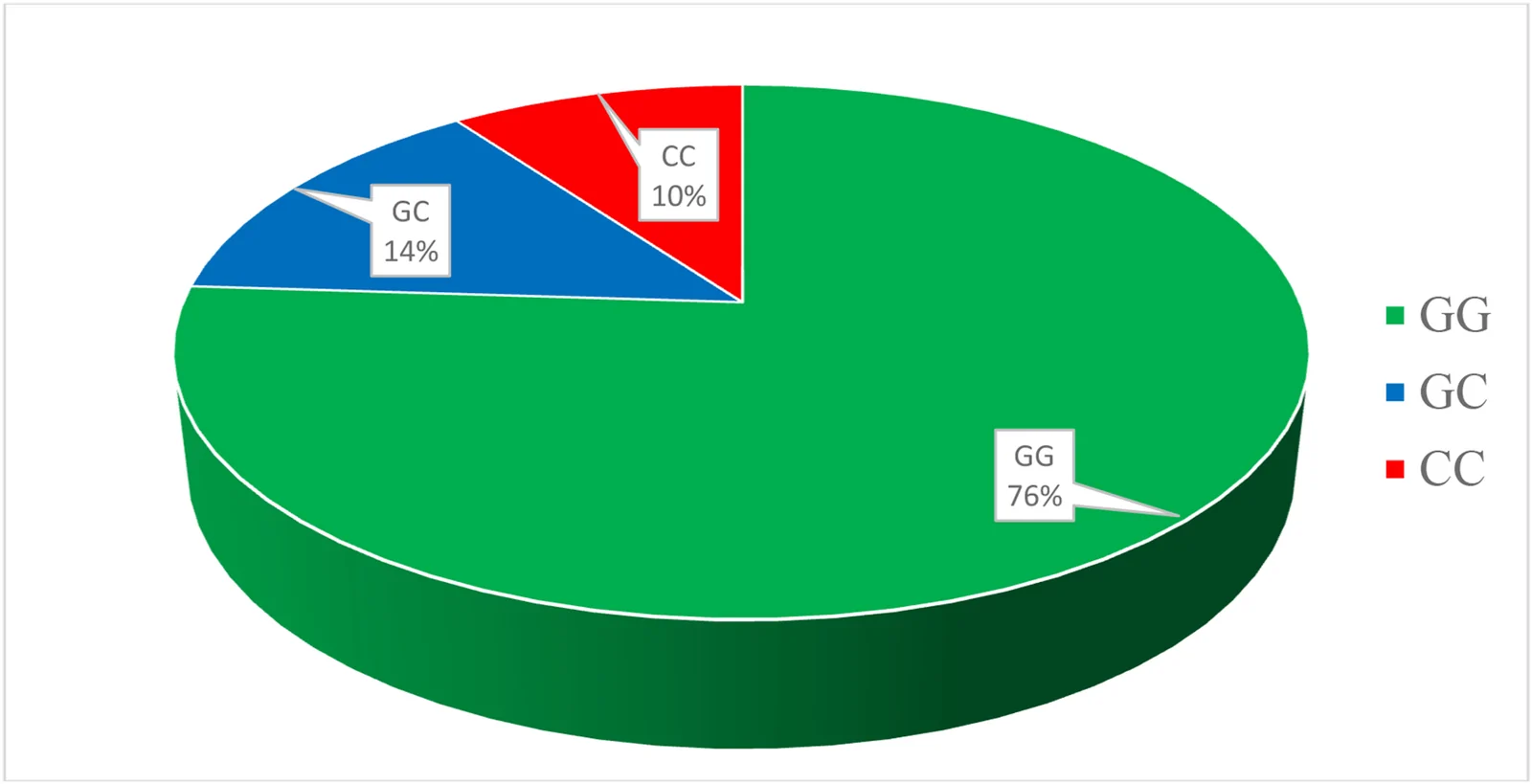
The prevalence of SLCO1B3 767G>C genotypes among breast cancer women.

The distinct bands of 337 bp indicated the SLCO1B3 767G>C (rs60140950) genotyping results. The size of this gene amplicon was determined based on comparisons with a DNA ladder ranging from 100 to 1000 bp, and the genotypes were categorized into three groups: the predominant wild type for allele G, the homozygous mutant type (CC) for allele C, and the heterozygous type (GC), as illustrated in Figure 1. The wild type was identified in approximately 76% of BC cases, whereas the heterozygous type (GC) and the homozygous mutant type (CC) were identified in roughly 14% and 10% of the cases, respectively, as illustrated in Figure 2. The distributions of SLCO1B3 767G>C (rs60140950) alleles and genotypes are presented in Table 2.

**Table 2:** The allele distribution of SLCO1B3 767G>C in the breast cancer women.

Genotype (N:150)	Frequency (%)	Allele	Frequency	Chi-square value	*P*-value
GG (wild type)	114 (76%)	G	0.83	38.09	<0.00001
GC (heterozygous type)	21 (14%)	C	0.17		
CC (homozygous type)	15 (10%)				

N: number of study cases.

#### Effects of SLCO1B3 767G>C genetic variation on BC markers

Serum levels of BC markers, specifically CA 15-3 and CEA, were assessed to examine the effects of different SLCO1B3 767G>C genotypes on the efficacy of paclitaxel. The results indicated that there were no significant differences among Iraqi women with BC carrying the GG, GC, and CC genotypes at *P* > 0.05, as shown in Table 3.

**Table 3:** The serum level of breast cancer markers according to SLCO1B3 767G>C genetic variation.

Parameters	Alleles of SLCO1B3 767G>C genotypes			*P*-value
	GG	GC	CC	
CA 15.3 (mIU/ml)	17.55 ± 17.05	20.5 ± 15.85	19.9 ± 23.9	0.563
CEA (mIU/ml)	2.31 ± 2.04	2.23 ± 4.89	3.55 ± 8.52	0.362

Data expressed as median ± IQR.

#### Effects of SLCO1B3 767G>C genetic variations on paclitaxel-related adverse effects

The elevated occurrence of paclitaxel-associated side effects, such as neutropenia, oral mucositis, and peripheral neuropathy, was evaluated to determine the potential effects of various SLCO1B3 767G>C genotypes on the safety profile of paclitaxel. The findings indicated that there were no significant correlations between SLCO1B3 767G>C genetic polymorphisms and the severity of paclitaxel-related side effects (neutropenia, oral mucositis, and peripheral neuropathy) at *P* > 0.05, as detailed in Table 4.

**Table 4:** The incidence and severity of paclitaxel-related adverse effects according to SLCO1B3 767G>C genetic variation.

Adverse effects		Alleles of SLCO1B3 767G>C genotypes			*P*-value
		GG	GC	CC	
Neutropenia	Score 1	20 (17.5%)	1 (4.8%)	4 (26.7%)	0.585
	Score 2	16 (14%)	5 (23.8%)	3 (20%)	
	Score 3	64 (56.2%)	12 (57.1%)	6 (40%)	
	Score 4	14 (12.3%)	3 (14.3%)	2 (13.3%)	
Oral mucositis	Score 0	28 (24.6%)	3 (14.3%)	1 (6.7%)	0.526
	Score 1	37 (32.4%)	7 (33.3%)	6 (40%)	
	Score 2	44 (38.6%)	9 (42.9%)	6 (40%)	
	Score 3	5 (4.4%)	2 (9.5%)	2 (13.3%)	
	Score 4	0 (0%)	0 (0%)	0 (0%)	
Peripheral neuropathy	Score 0	6 (5.2%)	1 (4.8%)	2 (13.3%)	0.864
	Score 1	55 (48.2%)	9 (42.8%)	6 (40%)	
	Score 2	42 (36.8%)	7 (33.3%)	6 (40%)	
	Score 3	3 (13.6%)	3 (9.7%)	7 (7.2%)	
	Score 4	0 (0%)	1 (3.2%)	2 (2.1%)	

Data expressed as No (%).

## Discussion

BC is among the most prevalent metastatic malignancies in Iraqi women, and a key aspect of its management and metastasis prevention is selecting effective and safe chemotherapy.^26^ Paclitaxel is widely utilized in chemotherapy for the treatment of BC, with significant efficacy, but resistance has developed in some patients.^27^ Multiple variables are considered to contribute to the diminished efficacy of paclitaxel, thereby resulting in suboptimal clinical responses. Several studies have suggested that route dysfunction, epigenetic alterations, and genetic variations could be prevalent contributing factors.^28^, ^29^, ^30^ OATP1B3 is a prominently expressed transporter encoded by the SLCO1B3 gene that facilitates the hepatic basolateral absorption of anionic substrates, playing a vital role in the systemic clearance of pharmaceuticals and toxins. One of these factors may influence the reaction to paclitaxel.^31^

The results obtained in the present study showed that the SLCO1B3 767G>C gene polymorphism was present in Iraqi women diagnosed with BC, where the homozygous GG genotype was found in 76% of cases, and the heterozygous GC and homozygous CC genotypes were observed in 14% and 10% of cases, respectively. The minor allele frequency was around 17% for the C allele compared with the reference major allele (G) in the study cohort. The incidence of SLCO1B3 767G>C is contingent upon ethnicity, where the mutant (CC) and heterozygous (GC) genotypes occur at rates of 2.2% and 31.1%, respectively, in the Caucasian-American population, whereas they are absent in African-, Chinese-, and Hispanic-American populations.^17^ Laitinen et al. (2010) demonstrated the lower prevalence of this variant in Finnish Caucasians, with a genotype distribution of GG = 76.6%, GC = 21.2%, and CC = 2.2%, corresponding to a C allele frequency of 12.8%. Thus, the frequency of the C allele is slightly higher in Iraqi women than that found in Caucasian-Americans and Europeans.^32^ Paclitaxel is a recognized substrate for the SLCO1B3 transporter, which facilitates its transport. SLCO1B3 gene polymorphisms can affect the cellular absorption of paclitaxel and its pharmacokinetic characteristics.^33^

The results obtained in the present study showed that the serum levels of BC markers (CA 15-3 and CEA) were within the normal ranges for most Iraqi women with BC, demonstrating that paclitaxel substantially inhibited metastases. CA 15-3 and CEA are routinely utilized for BC investigation, therapy monitoring, and progression assessment. CA 15-3 and CEA values of ≥20 U/mL and 3 μg/L, respectively, indicate that a malignancy responds poorly to therapy.^34^ The associations between the SLCO1B3 767G>C gene polymorphism and blood tumor markers (CA 15-3 and CEA) were not statistically significant (*P* = 0.563 and *P* = 0.362, respectively) among GG, GC, and CC carriers.

A study of a European Caucasian population found no correlations between prevalent SLCO1B3 polymorphisms (including 767G>C) and paclitaxel pharmacokinetics, indicating that these variants had little influence on the effects of paclitaxel within the cohort.^18^ The OATP1B3 transporter, encoded by the SLCO1B3 gene, is predominantly expressed at the basolateral plasma membrane in hepatocytes and is recognized as the most effective influx transporter for taxanes such as paclitaxel, but a clinical trial involving 449 women with BC or ovarian cancer showed that the 767G>C (rs60140950) polymorphism did not significantly affect paclitaxel clearance, the area under the receiver operating curve (AUC), or systemic exposure.^35^

The results obtained in the present study indicate that there were negligible correlations between the SLCO1B3 767G>C genetic polymorphism and the severity of paclitaxel-induced side effects. Another study found no significant associations between SLCO1B3 genotypes and paclitaxel-induced peripheral neuropathy in Spanish individuals.^36^ Paclitaxel causes numerous adverse effects, where neutropenia, oral mucositis, nausea and vomiting, alopecia, and peripheral neuropathy are the most prevalent.^37^ Previous studies examined the effects of various SLCO1B3 genetic variants on paclitaxel-induced neutropenia and showed that SLCO1B3 was associated with sensitivity to thrombocytopenia but not to neutropenia, suggesting paclitaxel penetration in megakaryocyte progenitors based on SLCO1B3 rather than in neutrophil progenitors.^38^ The SLCO1B3 767G>C genetic polymorphism (rs73241801) is not directly associated with taxane-induced peripheral neuropathy, where studies have obtained inconsistent findings regarding its influence, or demonstrated a correlation with overall prognosis rather than specific toxicities such as neurotoxicity. SLCO1B3 facilitates the transport of paclitaxel but the functional implications of 767G>C variations for paclitaxel clearance or toxicity are ambiguous, and have not been reliably validated in diverse patient cohorts.^39^

## Limitation of study

A notable limitation of this study was the low prevalence of the mutant CC genotype in the study population, thereby limiting the statistical power (<80%) for subgroup analyses. Therefore, it is important to collect a large sample size across many centers to validate possible associations.

## Conclusion

According to the present study, the SLCO1B3 767G>C genotype was significantly distributed among Iraqi women with BC, but there were no statistically significant associations between SLCO1B3 767G>C genetic variations and changes in BC markers (CA 15-3 and CEA) or the incidence of early paclitaxel-related adverse effects.

## Ethical approval

This study was approved by the scientific and ethical committee at Kerbala University, College of Pharmacy under project No: 2022HU9, and by the Ministry of Health of Iraq - Kerbala health department under project No: 433.

## Authors’ contributions

AB participated in the study's design and execution, analysis of the results, and preparation of the manuscript. AA and AH contributed to the review and editing of the study, and supervised the research. AS contributed to data collection and critically appraised the manuscript. All authors have critically reviewed and approved the final draft and are responsible for the content and similarity index of the manuscript.

## Source of funding

No financial support was provided for this study.

## Conflict of interest

The authors have no conflicts of interest to declare.

## References

[1] Kim J., Harper A., McCormack V., Sung H., Houssami N., Morgan E. (2025). Global patterns and trends in breast cancer incidence and mortality across 185 countries. Nat Med.

[2] Salih H.H., Abd S.Y., Al-Kaseer E., Al-Diwan J. (2024). Cancer in Iraq, general view of annual report 2022. J Contemp Med Sci.

[3] Al-Juhaishi A.M.R., Alaskari A.A.J., Nasser H.A. (2023). Correlation the severity of premenstrual symptoms, duration of breastfeeding, and incidence of breast cancer. Lat Am J Pharm.

[4] Chasib Z.M., Gaaib J.N. (2024). The association of five novel variants of TLR7 gene with some biochemical markers in breast cancer patients from Iraqi women. Karbala J Pharm Sci.

[5] Marino P., Mininni M., Deiana G., Marino G., Divella R., Bochicchio I. (2024). Healthy lifestyle and cancer risk: modifiable risk factors to prevent cancer. Nutrients.

[6] Hasan D. (2022). Diagnostic impact of CEA and CA 15-3 on chemotherapy monitoring of breast cancer patients. J Circ Biomarkers.

[7] Ying N., Liu S., Zhang M., Cheng J., Luo I., Jiang J. (2023). Nano delivery system for paclitaxel: recent advances in cancer theranostics. Colloids Surf B Biointerfaces.

[8] Zenjanab M.K., Alimohammadvand S., Doustmihan A., Kianian S., Oskouei BS., Mazloomi M. (2024). Paclitaxel for breast cancer therapy: a review on effective drug combination modalities and nano drug delivery platforms. J Drug Deliv Sci Technol.

[9] Kinnel B., Singh S.K., Oprea-Ilies G., Singh R. (2023). Targeted therapy and mechanisms of drug resistance in breast cancer. Cancers.

[10] Ye S., Chen S., Yang X., Lei X. (2024). Drug resistance in breast cancer is based on the mechanism of exocrine non-coding RNA. Discov Oncol.

[11] Alalawy A.I. (2024). Key genes and molecular mechanisms related to paclitaxel resistance. Cancer Cell Int.

[12] Hasan D.A.M., Sahib A.S., Al-Rokan A.A.H., Mohsin K.K. (2023). Study of the genetic polymorphisms of ABCB1 3435G> A in postmenopausal women breast cancer on paclitaxel chemotherapy. J Contemp Med Sci.

[13] Sousa-Pimenta M., Estevinho L.M., Szopa A., Basit M., Khan K., Armaghan M. (2023). Chemotherapeutic properties and side-effects associated with the clinical practice of terpene alkaloids: Paclitaxel, docetaxel, and cabazitaxel. Front Pharmacol.

[14] Aziz N.D., Abbood S.H., Al-Mayali A.H., Hadi N.R. (2023). aSSoCiatioN oF SolUtE CarriEr orGaNiC aNioN traNSPortEr 1B1 GENE PolyMorPHiSM witH rESPoNSE to atorvaStatiN aNd aSSoCiatEd MyoPatHy iN iraQi dySliPidEMia PatiENtS. Pol Merkur Lek.

[15] Hagenbuch B., Stieger B., Locher K.P. (2024). Organic anion transporting polypeptides (OATPs/SLCOs): pharmacology, toxicology, structure, and transport mechanisms. Pharmacol Rev.

[16] Anabtawi N., Drabison T., Hu S., Sparreboom A., Talebi Z. (2022). The role of OATP1B1 and OATP1B3 transporter polymorphisms in drug disposition and response to anticancer drugs: a review of the recent literature. Expert Opin Drug Metab Toxicol.

[17] Schwarz U.I., zu Schwabedissen H.E., Tirona R.G., Suzuki A., Leake B.F., Mokrab Y. (2011). Identification of novel functional organic anion-transporting polypeptide 1B3 polymorphisms and assessment of substrate specificity. Pharmacogenet Genom.

[18] Smith N., Marsh S., Scott-Horton T., Hamada A., Mielke S., Mross K. (2007). Variants in the SLCO1B3 gene: interethnic distribution and association with paclitaxel pharmacokinetics. Clin Pharmacol Therapeut.

[19] Gradishar W.J., Moran M.S., Abraham J., Aft R., Agnese D., Allison KH. (2022). Breast cancer, version 3.2022, NCCN clinical practice guidelines in oncology. J Natl Compr Cancer Netw.

[20] Limenh L.W., Tessema T.A., Simegn W., Ayenew W., Bayleyegn ZW., Sendekie AK. (2024). Patients’ preference for pharmaceutical dosage forms: does it affect medication adherence? A cross-sectional study in community pharmacies. Patient Prefer Adherence.

[21] Yanagihara F., Okura H., Ichikawa H., Shirakawa T., Pan Y., Tu B (2023). Development of an automated chemiluminescent immunoassay for cancer antigen 72–4 and the evaluation of its analytical performance. Pract Lab Med.

[22] Ba Y., Shi Y., Jiang W., Feng J., Cheng Y., Xiao L. (2020). Current management of chemotherapy-induced neutropenia in adults: key points and new challenges: committee of neoplastic supportive-care (CONS), China anti-cancer association committee of clinical chemotherapy, China anti-cancer association. Cancer Biol Med.

[23] Lalla R.V., Sonis S.T., Peterson D.E. (2008). Management of oral mucositis in patients who have cancer. Dent Clin.

[24] Zhang X., Chen W.W., Huang W.J. (2017). Chemotherapy-induced peripheral neuropathy. Biomedical reports.

[25] Kareem N.R., Mosa A.U., Al-Juhiashi A.M. (2025). Impact of CYP2A6 genetic polymorphism on letrozole efficacy in Iraqi women with polycystic ovary syndrome. J Taibah Univ Med Sci.

[26] Ali Salman R. (2023). Prevalence of women breast cancer. Cell. Mol Biomed Rep.

[27] Abouzeid H.A., Kassem L., Liu X., Abuelhana A. (2025). Paclitaxel resistance in breast cancer: current challenges and recent advanced therapeutic strategies. Cancer Treat Res Commun.

[28] Assunção H.C., Silva P.M., Bousbaa H., Cidade H. (2025). Recent advances in microtubule targeting agents for cancer therapy. Molecules.

[29] Li J., Yang F., Wei F., Ren X. (2017). The role of toll-like receptor 4 in tumor microenvironment. Oncotarget.

[30] Glaviano A., Singh S.K., Lee E.H.C., Okina E., Lam HY., Carbone D. (2025). Cell cycle dysregulation in cancer. Pharmacol Rev.

[31] van de Steeg E., van Esch A., Wagenaar E., Kenworthy K.E., Schinkel A.H. (2013). Influence of human OATP1B1, OATP1B3, and OATP1A2 on the pharmacokinetics of methotrexate and paclitaxel in humanized transgenic mice. Clin Cancer Res.

[32] Laitinen A., Niemi M. (2011). Frequencies of single-nucleotide polymorphisms of SLCO1A2, SLCO1B3 and SLCO2B1 genes in a Finnish population. Basic Clin Pharmacol Toxicol.

[33] Sun R., Ying Y., Tang Z., Liu T., Shi F., Li H. (2020). The emerging role of the SLCO1B3 protein in cancer resistance. Protein Pept Lett.

[34] Tarighati E., Keivan H., Mahani H. (2023). A review of prognostic and predictive biomarkers in breast cancer. Clin Exp Med.

[35] Apellániz-Ruiz M., Tejero H., Inglada-Pérez L., Sánchez-Barroso L., Gutiérrez-Gutiérrez G., Calvo I. (2017). Targeted sequencing reveals low-frequency variants in EPHA genes as markers of paclitaxel-induced peripheral neuropathy. Clin Cancer Res.

[36] Leskelä S., Jara C., Leandro-Garcia L., Martinez A., Garcia-Donas J., Hernando S. (2011). Polymorphisms in cytochromes P450 2C8 and 3A5 are associated with paclitaxel neurotoxicity. Pharmacogenomics J.

[37] Naeem A.G., Fawzi S.F., Elmorsi R.M., George M.Y. (2025). Paclitaxel-induced adverse effects: insights into multi-organ toxicities and molecular mechanisms. N Schmied Arch Pharmacol.

[38] Mbatchi L.C., Schmitt A., Thomas F., Cazaubon Y., Robert J., Lumbroso S. (2015). Polymorphisms in SLCO1B3 and NR1I2 as genetic determinants of hematotoxicity of carboplatin and paclitaxel combination. Pharmacogenomics.

[39] Guijosa A., Freyria A., Espinosa-Fernandez J.R., Estrada-Mena FJ., Armenta-Quiroga AS., Ortega-Treviño MF. (2022). Pharmacogenetics of taxane-induced neurotoxicity in breast cancer: systematic review and meta-analysis. Clin Transl Sci.

